# Multidisciplinary approach and anesthetic management of a surgical cancer patient with methylene tetrahydrofolate reductase deficiency: a case report and review of the literature

**DOI:** 10.1186/s13256-015-0662-0

**Published:** 2015-08-20

**Authors:** Marco MC Cascella, Manuela MA Arcamone, Emanuela EM Morelli, Daniela DV Viscardi, Viera VR Russo, Silvia SDF De Franciscis, Andrea AB Belli, Rosanna RA Accardo, Domenico DC Caliendo, Elena EDL De Luca, Barbara BDC Di Caprio, Francesco FDS Di Sauro, Giovanni GG Giannoni, Carmine CI Iermano, Maria MM Maciariello, Marcella MM Marracino, Arturo AC Cuomo

**Affiliations:** 1 Division of Anesthesia, Department of Anesthesia, Endoscopy and Cardiology, Istituto Nazionale Tumori “Fondazione G. Pascale” - IRCSS, Naples, Italy; 2 Division of Haematology-Oncology and Stem Cell Transplantation Unit, Istituto Nazionale Tumori “Fondazione G. Pascale” - IRCSS, Naples, Italy; 3 Department of Abdominal Oncology Surgery, Istituto Nazionale Tumori “Fondazione G. Pascale”- IRCSS, Naples, Italy; 4 Department of Surgery, Anesthesia, Emergency and Intensive Care, University of Naples “Federico II”, Naples, Italy

## Abstract

**Introduction:**

Hyperhomocysteinemia is a known risk factor for myocardial infarction, stroke, peripheral vascular disease, and thrombosis. Elevated plasma homocysteine levels have been demonstrated in patients with recurrent episodes or a single episode of thrombosis. Here we describe the development of cardiovascular disease as a complication of a surgical intervention in a patient with colorectal cancer and hyperhomocysteinemia.

**Case presentation:**

A 65-year-old Caucasian man complained of pain and constipation, attributed to previously diagnosed adenocarcinoma (stage IIB) of the hepatic flexure. An anamnestic investigation showed that he had undergone two surgical interventions. During both, he suffered thrombotic postoperative complications, a deep vein thrombosis of the upper extremity after the first operation and retinal vein occlusion after the second. He was diagnosed with hyperhomocysteinemia associated with a homozygous C677T mutation of the gene encoding the enzyme methylenetetrahydrofolate reductase. Our patient was initially treated with folic acid and high-dose B vitamins. On day 7 he underwent a right hemicolectomy. Anesthesia was performed with sevoflurane in 40% O_2_ and without the use of nitrous oxide. Postoperatively, our patient remained on folic acid and B vitamins and was without immediate or subsequent complications.

**Conclusions:**

Neoplastic disease and related surgery followed by the administration of chemotherapeutic drugs alter the hemostatic balance in cancer patients. Those suspected of also having a thrombophilic disease require a thorough laboratory diagnostic workup, including a molecular analysis aimed at identifying the genetic mutation responsible for the hyperhomocysteinemia, as indicated. The case described in this report highlights the importance of a multidisciplinary approach that includes expertise in peri-operative anesthesia, surgery, oncology, and hematology.

## Introduction

Clinical and epidemiological studies have shown that alterations in homocysteine (Hcy) metabolism play a role in atherosclerosis [1–3]. This would explain the approximately 20% of patients with atherosclerotic vascular disease but without any known cardiovascular risk factors, such as a family history, diabetes, smoking, and high blood pressure [4].

Hcy is a sulfur-containing amino acid that is formed during the methionine (MET) cycle. MET is an essential amino acid present in proteins of animal origin. Dietary MET is converted from adenosyltransferase methionine to S-adenosylmethionine (SAM). As the major donor of the methyl groups required in transmethylation reactions, SAM is critical to the synthesis of proteins, nucleic acids, creatinine, phospholipids, and neurotransmitters. The plasma level of Hcy is controlled by two distinct metabolic pathways. In the first, Hcy is degraded to cysteine, which is eliminated via the kidney; in the second, Hcy is converted to MET through a methylation reaction in which Hcy acquires a methyl group from either N5-methyltetrahydrofolate (MTHF) or from betaine and is then reconverted to MET. MET methylation is catalyzed by MET synthase using vitamin B12 as a cofactor and MTHF as the methyl donor. MTHF is formed from folic acid by the enzyme N5, N10 methylenetetrahydrofolate reductase (MTHFR).This MET recovery pathway contributes to regulating the serum Hcy concentration, and alterations therein underlie the development of Hcy-related atherosclerosis [5].

Under normal conditions, there is a close balance between the formation and elimination of Hcy, with approximately 50% being methylated to MET. In the presence of excess MET, a larger amount is converted to cysteine via a transulfuration reaction. If the rate of cellular Hcy formation is faster than the rate of Hcy consumption, the excess is released into the circulation, leading to hyperhomocysteinemia (HHcy).

A possible cause of HHcy is the presence of genetic abnormalities that affect both the sulfuration reaction and the methylation of Hcy to MET. Two known MTHFR polymorphisms are associated with the reduced activity of the enzyme and therefore with increased levels of Hcy: the C677T polymorphism and the A1298C polymorphism. Of these, the former is considered the most important genetic determinant of Hcy concentrations.

MTHFR mutations are relatively common. For example, homozygosity for MTHFR C677T is present in 0–3% of African Americans and in 9–11% of Caucasian Americans. These mutations may remain undetected but they are also associated with an increased incidence of thrombotic diseases. However, in rare cases defects in MTHFR cause severe increases in plasma Hcy, leading to gait abnormalities, seizures, and psychiatric manifestations. Here we describe the development of cardiovascular disease as a complication of a surgical intervention in a patient with colorectal cancer and hyperhomocysteinemia.

## Case presentation

A 65-year-old Caucasian man complained of pain and constipation related to adenocarcinoma (stage IIB) involving the hepatic flexure. He had a long-standing history of hypertension, but it was well controlled with a beta blocker. An anamnestic investigation showed that he had been operated on twice: approximately 10 years earlier he had undergone surgical repair of an inguinal hernia, and 2 months earlier, a transurethral resection for a papillomatous lesion of the bladder. Following both surgeries, he had symptoms indicative of thrombotic complications. The first operation was followed by the rapid onset of postoperative pain in the left arm, with swelling and redness. These symptoms lasted for approximately 1 month and were treated with antibiotics and low-molecular-weight heparin. His symptoms suggested a deep vein thrombosis (DVT) of the upper extremity, a suspicion confirmed by the finding of prominent veins in our patient’s left arm. After the second intervention, he experienced a severe loss of vision in his left eye, attributed to retinal vein branch occlusion (RVO).

Our patient’s prior history was consistent with a hemostatic disorder. Before undergoing surgery for his cancer-related complaints, a complete screening for risk factors of thrombosis was performed. The levels of C and S proteins were in the normal range; his serum Hcy level was 25μmol/L. A subsequent molecular analysis showed homozygosity for the MTHFR C677T mutation. He did not have the Leiden variant of factor V. Treatment with folic acid (25mg/day intramuscularly (i.m.)), cocarboxylase (38mg/day, i.m.), pyridoxine hydrochloride (300mg/day, i.m.), hydroxocobalamin (5000μg/day, i.m.), and antiplatelet therapy with low-dose aspirin was started. After his serum Hcy level had decreased to 18μmol/L, he underwent a right hemicolectomy. Low-dose aspirin therapy was maintained. Anesthesia was performed with sevoflurane in 40% O_2_, without the use of nitrous oxide (N_2_O).

The operative course was uneventful and there were no immediate or late complications as determined in a physical examination, molecular laboratory analysis, blood test, and Doppler ultrasound screening of the upper and lower extremities performed 7 days, 15 days, 1 month, 3 months, and 6 months postoperatively. His serum Hcy levels were 13μmol/L, 11.5μmol/L, 10.4μmol/L, 9.8μmol/L, and 8.7μmol/L, respectively.

Antithrombotic therapy after surgery consisted of 100mg of intravenous acetylsalicylic acid every 24 hours starting 12 hours after the end of the surgery. On day 3, our patient resumed oral therapy with low-dose aspirin. The administration of B vitamins continued with high doses during the first 7 postoperative days, followed by oral folic acid (15mg/day) and hydroxocobalamin (5μg/day) for maintenance.

## Discussion

The suspicion that our patient had a genetically determined hemostatic alteration arose when his anamnesis included thrombotic complications after previous surgeries.

DVT of the upper extremity is a rare event, accounting for only 1-4% of DVTs [6]. This complication is usually associated with the placement of a central venous catheter [7]; however, this was not the case in our patient, in whom the device was never implanted. His report of RVO led us to investigate the clinical aspects and pathogenesis underlying his medical history. HHcy was recently identified as an emerging risk factor for RVO. Although the main risk factors are hypertension and glaucoma, elevated serum Hcy levels are a more frequent occurrence than other known risk factors for cardiovascular diseases (for example, diabetes, dyslipidemia, smoking, hyperviscosity syndrome, and vasculitis) in patients with RVO. This association is supported by the high frequency of homozygosity for the C677T MTHFR gene polymorphism in patients with RVO [8, 9].

Once the diagnosis of thrombophilic disease was established, an appropriate peri-operative protocol was chosen. Surgery and its related stress can alter the patient’s hemostatic balance; this is especially the case in cancer patients. Indeed, the increased risk of thromboembolism in cancer patients has long been recognized, and it has been attributed to the complex state of hypercoagulability. Chemotherapy enhances the risk of thrombosis by causing endothelial damage.

Cancer-related thrombosis is the second leading cause of death (after cancer itself) in cancer patients and is accompanied by a marked worsening of the prognosis [10]. Therefore, in this cohort of patients, it is of fundamental importance to promptly identify additional factors leading to an increased thrombophilic risk, as this will insure a timely intervention aimed at minimizing thrombotic complications during the postoperative period.

Peri-operative changes in hemostasis have a multifactorial origin but may also be secondary to medications, including anesthetic agents. In fact, most anesthetics, including those administered intravenously, volatile anesthetics, and local anesthetics, have been linked to the inhibition of platelet function, albeit to varying extents [11, 12].

The anesthetic agent with the greatest impact on folate metabolism is N_2_O, which deactivates cobalamin via an oxidation reaction and causes an irreversible block of MET synthase, located at the confluence of Hcy methylation and the folate cycle. This results in a sustained increase in plasma Hcy concentrations and in the absence of biologically active folate (‘folate trapping’) that can be used for the conversion of Hcy to MET [13]. Therefore, N_2_O-induced MET synthase inhibition in patients with MTFR mutations can cause severe hematological and neurological effects and the use of N_2_O during anesthesia should therefore be avoided in this cohort.

Over the last 30 years, more than a dozen clinical cases have been published in which N_2_O anesthesia led to neurological complications, including myelopathy, peripheral neuropathy, and hemiparesis, caused by a lack of MET in the brain [14, 15].

The neurological damage caused by N_2_O is often accompanied by hematological abnormalities, such as megaloblastic anemia and bone marrow disorders. Selzer *et al.* [16] proposed the ‘double-hit’ mechanism to explain the neurological deterioration and death of a child anesthetized twice with N_2_O. This hypothesis links HHcy and the N_2_O-induced inhibition of folate metabolism with a defect in the MTHFR gene. Our case study suggests that the ‘double-hit’ hypothesis is also applicable to patients with a cobalamin or folate deficiency who are exposed to N_2_O, for example, patients with other MTHFR gene polymorphisms [17]. Thus, in patients in whom altered Hcy metabolism is suspected, a more in-depth molecular diagnosis and, if the surgical intervention cannot be postponed, the avoidance of N_2_O-containing anesthesia are recommended.

Badner *et al*. described an association between Hcy and a significant increase in the incidence and duration of postoperative ischemia in patients receiving N_2_O-containing anesthesia for carotid endarterectomy [18].

Although the inhibition of cobalamin and HHcy after N_2_O administration is transient, lasting only for a few days [19], the effect coincides with the postoperative period, during which the patient is particularly vulnerable, as evidenced by the very high risk of serious cardiovascular complications on postoperative days 2 and 3 [20, 21]. In addition, Hcy levels seem to correlate with the duration of N_2_O exposure. The preoperative administration of B vitamins can prevent the effects induced by N_2_O [22].

In the ENIGMA study, patients undergoing noncardiac surgery who were at low cardiovascular risk and exposed to N_2_O had an unexpectedly high number of myocardial infarctions [23]. Recent studies have also shown that patients homozygous for the MTHFR mutation who receive N_2_O anesthesia have higher postoperative plasma Hcy levels than patients who are heterozygous for the mutation or carry the wild-type gene [24].

In contrast to previous work, Nagele *et al*. [25] suggested that neither N_2_O-induced HHcy nor MTHFR gene polymorphisms are associated with postoperative cardiac events. This implies a minor role for genetic influences in the response to anesthesia and that the prophylactic use of B vitamins would thus be effective in reducing anesthesia-induced increases in Hcy levels but not peri-operative cardiac events.

To determine the true impact of N_2_O on cardiovascular events requires a large and rigorous study. The results of ENIGMA II, in which 7000 patients undergoing major surgery were enrolled, supports the safety profile of N_2_O for use in major noncardiac surgery, although these patients had normal Hyc values [26].

Another consideration related to the MTHFR mutation is its association with colorectal cancer and its interference with chemotherapy. MTHFR plays an important role in folate metabolism, which has been implicated in carcinogenesis because of the folate dependence of methylation, repair, and DNA synthesis. In previous work, we analyzed the relationship between C677T and A1298C MTHFR gene polymorphisms and the biological, pathological, genetic, and epigenetic characteristics of several different tumor types and their response to chemotherapy with 5-fluorouracil. The aim of that study was to determine the contribution of the MTHFR genotype to the susceptibility to colorectal cancer and the response to treatment [27–29]. While some authors have reported the protective effect of the allelic variant C677T in the development of several cancers [27, 28], the meta-analysis of Pu *et al*. showed an association between this polymorphism and an increased susceptibility to ovarian cancer in the Asian population [29]. In addition, the MTHFR A1298C polymorphism is reportedly associated with a high risk of colon cancer [30], but according to the meta-analysis of Zhao *et al*. [31] both the MTHFR 677T and the 1298C alleles are associated with a low risk of colorectal cancer. This finding further emphasizes the uniqueness of our case because we found the C677T mutation in our patient. Both polymorphisms may also be influenced by independent factors that in turn determine the response to 5-fluorouracil. Thus, MTHFR genotyping may guide the selection of the optimal treatment regimen [32].

## Conclusions

Patients with neoplastic disease frequently undergo surgery, followed by the administration of chemotherapy. Because these three factors are known to alter hemostatic balance, the suspicion of a thrombophilic disease in a cancer patient must include a thorough laboratory diagnostic evaluation. If elevated serum Hcy levels are found, a complete screening, including a molecular analysis aimed at identifying the type of genetic mutation, is warranted.

Our case study also emphasizes the importance of a multidisciplinary approach. Multicenter studies have sought to quantify the cardiovascular complications associated with the use of N_2_O in patients with HHcy. The ENIGMA II study showed that N_2_O does not increase the risk of death and cardiovascular complications or surgical site infection in patients undergoing major noncardiac surgery. However, these results are valid only for patients with normal Hyc values. Whether MTHFR gene polymorphisms are associated with an increased risk of N_2_O-induced cardiac events remains to be determined. In the meantime, however, the use of N_2_O in these patients should be avoided and the thrombophilic effect of HHcy minimized using acetylsalicylic acid prophylaxis, folic acid, and B vitamins. Finally, our results have oncological implications, as they suggest that MTHFR polymorphisms influence the susceptibility to colorectal cancer.

## Consent

Written informed consent was obtained from the patient for publication of this case report and any accompanying images. A copy of the written consent is available for review by the Editor-in-Chief of this journal.
