# Erratum to: Multidisciplinary approach and anesthetic management of a surgical cancer patient with methylene tetrahydrofolate reductase deficiency: a case report and review of the literature

**DOI:** 10.1186/s13256-015-0706-5

**Published:** 2015-09-17

**Authors:** M Cascella, M Arcamone, E Morelli, D Viscardi, V Russo, S De Franciscis, A Belli, R Accardo, D Caliendo, E De Luca, B Di Caprio, F Di Sauro, G Giannoni, C Iermano, M Maciariello, M Marracino, A Cuomo

**Affiliations:** 1 Division of Anesthesia, Department of Anesthesia, Endoscopy and Cardiology, Istituto Nazionale Tumori “Fondazione G. Pascale” - IRCSS, Naples, Italy; 2 Division of Haematology-Oncology and Stem Cell Transplantation Unit, Istituto Nazionale Tumori “Fondazione G. Pascale” - IRCSS, Naples, Italy; 3 Department of Surgery, Anesthesia, Emergency and Intensive Care, University of Naples “Federico II”, Naples, Italy; 4 Department of Abdominal Oncology Surgery, Istituto Nazionale Tumori “Fondazione G. Pascale”- IRCSS, Naples, Italy

The original version of this article [1] unfortunately contained a mistake. The presentation of the author names is incorrectly marked up and therefore it is presented incorrectly in the HTML version of this article. The corrected author list is given below:

Cascella M, Arcamone M, Morelli E, Viscardi D, Russo V, De Franciscis S, Belli A, Accardo R, Caliendo D, De Luca E, Di Caprio B, Di Sauro F, Giannoni G, Iermano C, Maciariello M, Marracino M, Cuomo A.

The original article was corrected accordingly.
